# Hydrogen peroxide pretreatment assisted phytoremediation of sodium dodecyl sulfate by *Juncus acutus* L

**DOI:** 10.1186/s12870-022-03984-0

**Published:** 2022-12-16

**Authors:** Amany H. A. Abeed, Suzan A. Tammam, M. T. El-Mahdy

**Affiliations:** 1 Department of Botany and Microbiology, Faculty of Science, Assiut University, Assiut, 71516 Egypt; 2 Department of Pomology, Faculty of Agriculture, Assiut University, Assiut, 71526 Egypt

**Keywords:** Hydrogen peroxide, *Juncus acutus* L., Phytoremediation, Sodium dodecyl sulfate, Surfactants

## Abstract

**Background:**

Sodium Dodecyl Sulfate (SDS) an anionic surfactant pollutant has emerged as a serious hazard to the aquatic and terrestrial environment. Due to physical and chemical methodological difficulties for SDS removal, phytoremediation techniques are efficient alternative strategies to tackle such adversities. *Juncus acutus* L. (*J. acutus*) is a pioneer wetland species that has been recently exploited for phytoremediation purposes. To our knowledge, the role of exogenous hydrogen peroxide (H_2_O_2_), in improving the phytoextraction of SDS has not been examined yet. In this study, pretreatment foliar spray of H_2_O_2_ (15 mM) combined with two levels of SDS (50 and 100 ppm) in water culture was evaluated to remove SDS contamination and add value to the phytoremediation process.

**Results:**

The outcomes revealed that *J. acutus* has considerable translocation and bioaccumulation abilities for SDS and can be utilized as an appropriate hyperaccumulator in SDS-contaminated sites. However, the involvement of H_2_O_2_ extended phytoremediation capacity and successive removal of SDS. H_2_O_2_ significantly assisted in increasing SDS remediation via more accumulation in *J. acutus* tissues by 29.9 and 112.4% and decreasing SDS concentration in culture media by 33.3 and 27.3% at 50 and 100 ppm SDS, respectively. Bioaccumulation factor (BCF) increased by 13.8 and 13.2%, while translocation factor (TCF) positively maximized by 82.4 and 76.2% by H_2_O_2_ application at 50 and 100 ppm SDS, respectively. H_2_O_2_ pretreatment could drive the decline in biochemical attributes in SDS-affected plants by modulating stress tolerance indices, pigments, water relations, proline content, enzymatic activities, and further, reduced oxidative stress in terms of electrolyte leakage, cellular H_2_O_2_, malondialdehyde (MDA) accumulation.

**Conclusions:**

H_2_O_2_ could play a potential role in maximizing phytoremediation capacity of SDS by *J. acutus* in polluted sites.

## Background

In the intervening days, one of the most critical threatens to plant life and biosphere is the emerging surfactant pollutants. Surfactants are chemically synthesized products mostly derived from petroleum compounds [1] and characterized by their active properties in reducing surface tension or interfacial tension between two heterogeneous phases, thus have been used in massive applications of life sectors, ranging from food industries, pharmaceuticals, agrochemicals, and households [2, 3]. More crucially, the production of these surfactants is rapidly growing and is expected to exceed 50 billion dollars within few years due to the high demand of surfactant products like hand sanitizer and disinfectants during COVID-19 pandemic [4]. Surfactants are classified into anionic, cationic, nonionic, and amphoteric composites according to the electrolytic charge of the hydrophilic group [5]. Surfactants contain a polar head group attached with nonpolar hydrocarbon tail and being highly hydrophobic accelerate their pernicious diffuse in marines and surrounding environments [6]. Currently, it was shown that about 60% of surfactant residues contaminate the aquatic sides in significant concentrations [7]. Releasing wastes polluted with large amounts of synthetic surfactants into the water surfaces and nearby agricultural soils jeopardizes the plant community and ecosystem [8].

Sodium dodecyl sulfate (SDS, molecular formula: C_12_H_25_SO_4_Na, and molecular weight: 288.38 g/mol with hydrophobic hydrocarbon chain of 12 carbon atoms); is a type of negative charged anionic surfactant integrated in almost everyday products such as household cleaners, domestic detergents, and cosmetics due to its micellization behavior [8]. It is the most common surfactant extensively utilized in industries for its great emulsifying and fizzing qualities in cost-effective manner. SDS and anionic surfactants can change macromolecules structure and induce disfunction by binding to DNA, enzymes and peptides [9]. Moreover, they bind to plant cell wall molecules such as proteins and phospholipids and consequently alter membrane rigidification and impair its biological function [10]. Recent ecotoxicological studies proved that by continuous evoke of surfactants into the environment in heightened levels, the accumulation of SDS can induce oxidative burst in plants which may devastate cellular redox homeostasis and consequently physiological and biochemical complexes [11]. This eventually exacerbates plant dynamics growth and concomitant humane health through food chain. A crucial question is how plants can deal with all pollution burdens such as surfactants, particularly when combined with other problematic issues restricting plant growth such as soil salinization or alkalinization.

As the exposure of plants to SDS and other pollutants become frequent and a contaminant concern, The World Health Organization (WHO) has set the optimum permissible level of surfactant in water supplies not to exceed 0.2 mg/L [12] however, surfactant was formerly detected to exceed 400 mg/L in wastewater from manufacturing industries [13]. Thus, the current legislations require monitoring the acute toxic effect of the micropollutants to protect the environment and humane safety. It is important to evaluate the effects of pollutant type and concentration on plants performance and treat hazardous pollution on SDS-rich soils where plants grow.

In this regard, innovative strategies have been introduced to manage the severity of pollutant noxiousness including scavenging or removal by using different approaches. Among these methods, H_2_O_2_ has gained an increasing attention as a promising cytoprotective motivator toward multi-tolerance adaption mechanisms such as excess temperatures, drought, salinity, heavy metals, light, and UV stresses in numerous plant species [14–16]. In conserved plant systems, the accumulation of reactive oxygen species (ROS) is well known to be correlated with various cellular metabolic reactions under stressful conditions. Overproduction of ROS compartments like H_2_O_2_ can disrupt the biochemical and physiological pathways in multiple sites within the plant cell, which can lead to permanent cell rupture and programmed cell death [17, 18]. Importantly and in contrary to the classical concepts, plants have progressed several mechanisms to switch ROS signaling components under certain low levels to regulate wide variety of plant pathways, including cell growth and development, and balance adaptive responses to environmental stresses [19, 20]. H_2_O_2_ is a stable ROS product and is involved as a signaling molecule in regulating vital primary and secondary metabolic pathways under normal levels. These pathways include seed germination, shoot and root differentiation, development as well as, acclimation and guard cell signaling under normal levels [21].

In contaminated areas, of equal or higher importance to regular decontamination physical and chemical procedures, phytoremediation is well-established approach for depollution [22, 23]. Phytoremediation based on employing certain plant species possess a high capacity to store pollutants in their organelles with improved tolerance to their toxicity is an in situ safe cleanup route. The widespread exploitation of phytoremediator plants is seems to be mainly allied to their superior advantages compared to other plants. These advantages include plant proficiency in bioaccumulating pollutants; acting as phytostabilizers or phytoextractor, rush growth rate, eco-friendly to cover large areas in considerable economic value, and the inhibition of secondary effluence [24, 25]. By screenings phytoremediation plant species and ecotypes, wetland halophytes *Juncus* sp. (Juncaceae family) have been the interest of scientific research in recent years [26]. *Juncus* species have been identified by their great potential adaptative features to uptake and accumulate different contaminants in their biomass. *Juncus acutus* L. halophyte was selected for the purpose of this study because of the natural thrive of the specie in shorelines, dunes, lagoons, and salt marshes near the eutrophicated sites where industrial effluents and wastewater are abundant. The plant is accrediting for its potential hyperaccumulation of excess metals including zinc (Zn), cadmium (Cd), lead (Pb), and arsenic (As) [27–30] and petrogenic products such as diesel [31]. Thereby, *J. acutus* is considered elite contestant for the phytoremediation objectives of specific pollutant. To date, rare published literatures spotlight the transport-based remedial strategy of *J. acutus* in removing surfactants contamination within its tolerable limits and the improved role of H_2_O_2_ in promoting phytoremediation efficiency. Precisely, we aimed to (a) asses the phytoremediation value added capacity of *J. acutus* to eliminate SDS contamination at different functional behaviors across diverse levels of exposure, (b) enlighten the holistic role of H_2_O_2_ in promoting phytoremediation toward SDS toxicity by observing the underneath changes in morphological, physiological, and biochemical responses in individuals of *J. acutus.* For best of our knowledge, this manuscript is the first to investigate the interactive effects of concurrently SDS hazard toxicity and the removal mechanism by *J. acutus* phytoremediator with the assessment of H_2_O_2_. The high removal efficiency of SDS by *J. acutus* remediator provides informative insights for valuable bioindicator of SDS pollution level in a contaminated habitat. Implement of H_2_O_2_ with phytoremediators could further improve treatment efficiency. With the development of phytoremediation technology, these co-application strategies could help much progress for the rehabilitation of polluted agro- and/or aqua-systems.

## Results

### Phytoremediation parameters

We hypothesized that H_2_O_2_ play a central role in improving *J. acutus* L. remediation capacity for removing SDS contamination in plant environment by inducing specific physiological and biochemical changes in plant cellular responses. To investigate this hypothesis, H_2_O_2_ (15 mM) pretreatment was applied in the experiment separately or with two levels of SDS (50 or 100 ppm) in the culture media and relevant data are presented in Table 1. After two weeks of exposure to SDS (50 and 100 ppm) individually or combined with H_2_O_2_, data revealed that *J. acutus* has the ability to accumulate high levels of SDS equal to 87 and 105 μg/g FW at 50 and 100 ppm SDS, respectively. It was also noticed that application of H_2_O_2_ significantly assisted in increasing SDS remediation via more accumulation in *J. acutus* tissues by 29.9 and 112.4% and decreasing SDS concentration in culture media by 33.3 and 27.3% at 50 and 100 ppm SDS, respectively. Regarding bioaccumulation factor (BCF) and translocation factor (TCF), it was observed that values of BCF and TCF were less in plantlets without application of H_2_O_2_. However, pretreatment with H_2_O_2_ promoted the accumulation of SDS in *J. acutus* roots and leaves. BCF showed marked increase by 16 and 15.2%, while TCF positively increased by 82.4 and 76.2% at 50 and 100 ppm SDS, respectively. Importantly, 63 and 65% of SDS removal from the culture media was achieved when plants pretreated with H_2_O_2_ in response to 50 and 100 ppm SDS, respectively. These results revealed that exogenous H_2_O_2_ can promote phytoremediation potential of SDS by *Juncus* plants.

**Table 1:** Effect of sodium dodecyl sulfate (SDS) and hydrogen peroxide (H_2_O_2_) foliar spraying on SDS content in plant (μg/g FW), media (ppm), bioaccumulation factor (BCF), translocation factor (TCF), and % removed SDS in solution culture with increasing SDS concentrations (0, 50 and 100 ppm) treated with or without 15 mM H_2_O_2_

Treatment	SDS in plant (μg/g FW)	SDS in media (ppm)	BCF	TCF	% Removed SDS
Control	0.0	0.0	0.0	0.0	0.0
H_2_O_2_	0.0	0.0	0.0	0.0	0.0
50 ppm SDS	87 ± 1.1^d^	6 ± 0.4^c^	2.5 ± 0.1^d^	0.17 ± 0.001^c^	46 ± 1.1^c^
SDS50 + H_2_O_2_	113 ± 3.4^b^	4 ± 0.5^d^	2.9 ± 0.2^c^	0.31 ± 0.002^a^	75 ± 1.6^a^
100 ppm SDS	105 ± 2.1^c^	11 ± 0.9^a^	3.3 ± 0.11^b^	0.21 ± 0.001^b^	40 ± 1.8^d^
SDS100 + H_2_O_2_	223 ± 5.4^a^	8 ± 0.6^b^	3.8 ± 0.21^a^	0.37 ± 0.002^a^	66 ± 1.5^b^

^a,^ ^b,^ ^c,^ ^d^Different letters denote significant differences (*P* > 0.05)

### Characteristics of *J. acutus* growth stress indices

**Fig. 1:**
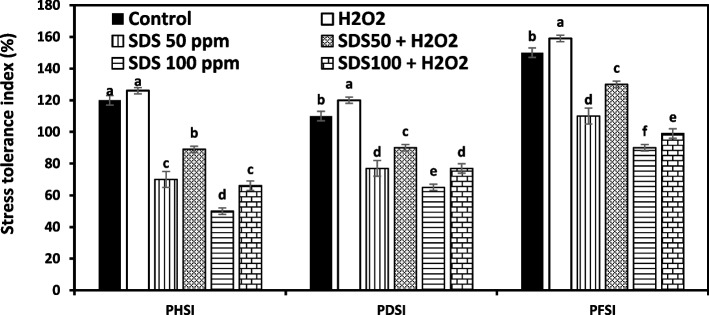
Effect of sodium dodecyl sulfate (SDS) and hydrogen peroxide (H_2_O_2_) foliar spraying on plant height stress tolerance index (PHSI), plant dry matter stress tolerance index (PDSI), and fresh matter stress tolerance index (PFSI) of *Juncus acutus* plantlets grown in solution culture with increasing SDS concentrations (0, 50 and 100 ppm) treated or not with 15 mM H_2_O_2_. Values are the means of four replications ± SE. Variants bearing the different letters donate statistically significant at *P* > 0.05

Plant growth parameters in terms of stress tolerance index of plant height (PHSI), Dry matter (PDSI), and fresh matter (PFSI) of *J. acutus* were measured. Plants were deleteriously affected by SDS treatment when compared to controls and the symptoms intensified with rising SDS level in the culture medium. Relative to SDS free group, PHSI, PDSI and PFSI of *J. acutus* reduced by 41.7, 30, and 26.6%, respectively at the lower treatment level of SDS (50 ppm) and by 58.3, 40.9, and 40%, respectively at the higher treatment level of SDS (100 ppm). However, when foliar sprayed H_2_O_2_ (15 mM) was applied, the respective reduction was only 25.8, 18.2, and 13.3% at 50 ppm SDS and 45, 30, and 34% at 100 ppm SDS. Exogenous spraying of H_2_O_2_ improved growth stress indices characteristics by reducing the inhibitory effects of SDS at both levels of stress showing that H_2_O_2_ obviously ameliorated SDS toxicity symptoms in *J. acutus* (Fig. 1)*.*

### Physiological and biochemical analysis

#### Photosynthetic pigments

**Fig. 2:**
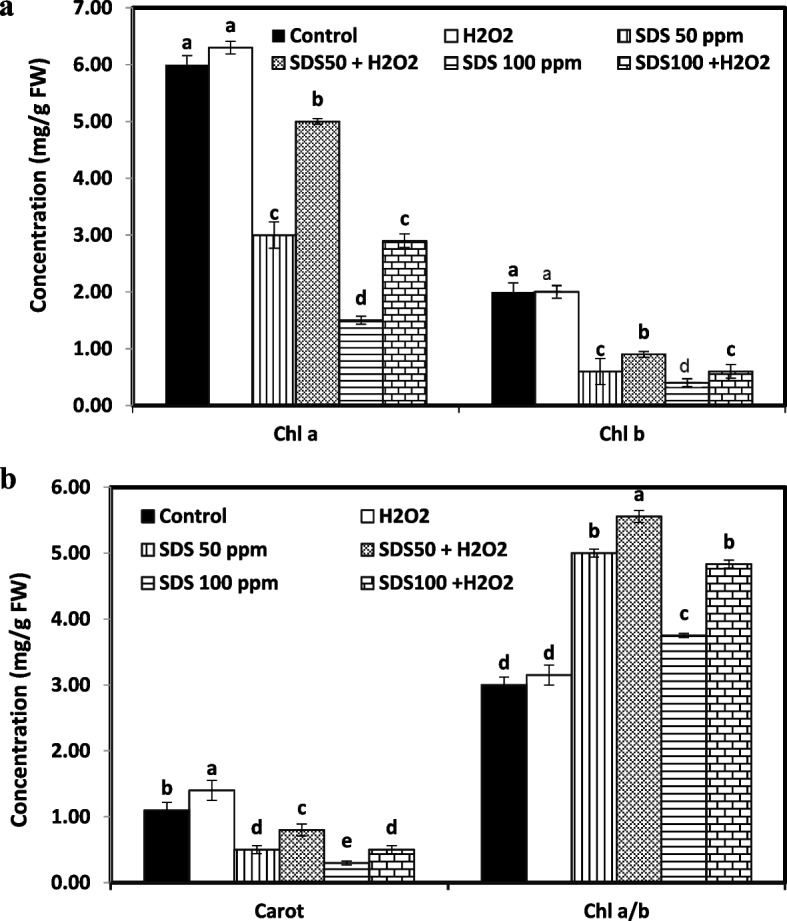
Effect of sodium dodecyl sulfate (SDS) and hydrogen peroxide (H_2_O_2_) foliar spraying on photosynthetic pigments; chlorophyll a (Chl a), chlorophyll b (Chl b), carotenoids (Carot) and Chl a/ b of *Juncus acutus* plantlets grown in solution culture with increasing SDS concentrations (0, 50 and 100 ppm) treated or not with 15 mM H_2_O_2_. Values are the means of four replications ± SE. Variants bearing the different letters donate statistically significant at *P* > 0.05

SDS showed an adverse effect on leaf pigment content. After SDS exposure, a significant drop in Chl a, Chl b, and carotenoids quantities was noted as compared to SDS- free treatment (Fig. 2). Compared to control, the highest level of SDS (100 ppm) resulted in 75, 80, and 72.7% decline in Chl a, Chl b, and carotenoids, respectively. With the addition of H_2_O_2_, the decrease in Chl a, Chl b, and carotenoids content was inhibited to 51.6, 70, and 54.5%, respectively. Under the lower level of SDS (50 ppm), the reduction was 50, 70, and 54.5% in Chl a, Chl b, and carotenoids. The combined treatment of H_2_O_2_ and SDS (50 ppm) induced only 16.6, 55, 27.2% reduction in the contents of Chl a, Chl b, and carotenoids, respectively. Data shows that application of H_2_O_2_ along with SDS significantly augmented leaf pigment contents as compared to respective SDS delivered plants without H_2_O_2_ application. No clear differences between H_2_O_2_ treated and untreated plants in chlorophylls content, but the variations were obvious in carotenoids content.

#### Gas exchange attributes

**Fig. 3:**
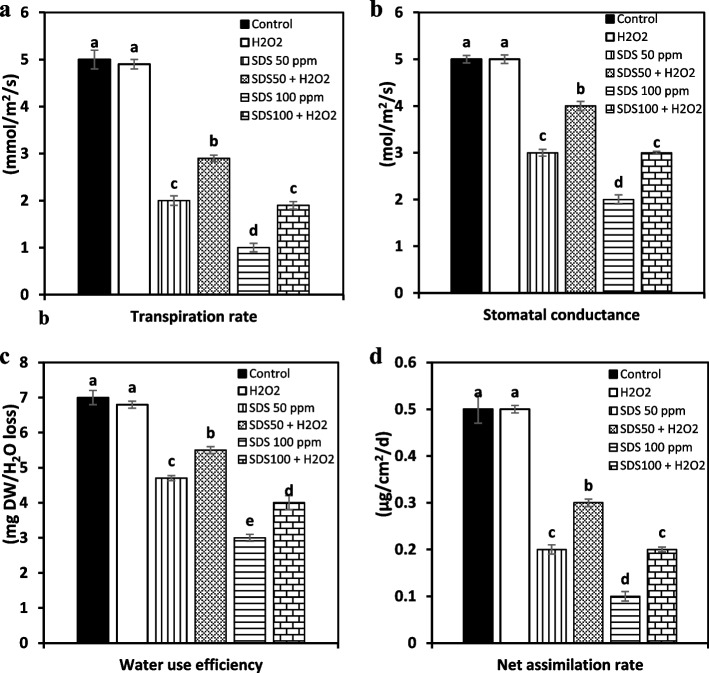
Effect of sodium dodecyl sulfate (SDS) and hydrogen peroxide (H_2_O_2_) foliar spraying on transpiration rate (**a**), stomatal conductance (**b**), water use efficiency (**c**), and net assimilation rate (**d**) of *Juncus acutus* plantlets grown in solution culture with increasing SDS concentrations (0, 50 and 100 ppm) treated or not with 15 mM H_2_O_2_. Values are the means of four replications ± SE. Variants bearing the different letters donate statistically significant at *P* > 0.05

Compared with the respective SDS- treated plants, H_2_O_2_ treatment evidently improved gas exchange attributes including transpiration rate, stomatal conductance, water use efficiency and assimilation rate and the improvement in these parameters was dose dependant. H_2_O_2_ pre-supply developed a noteworthy increase in transpiration rate from 2.0 to 2.9 and from 1.0 to 1.9 mmol/ m^2^/ s under 50 and 100 ppm SDS, respectively. Similarly, escalation in stomatal conductance performance increased from 3.0 to 4.0 and from 2.0 to 3.0 mol/m^2^/s as result of exposure to 50 and 100 ppm SDS, respectively. Moreover, water use efficiency recorded increased values from 4.7 to 5.5 and from 3.0 to 4.0 mg DW/H_2_O loss at 50 and 100 ppm SDS, respectively. Finally, Net assimilation rate was upgraded from 0.2 to 0.3 and from 0.1 to 0.2 μg/cm^2^/d in response to 50 and 100 ppm SDS, respectively. Gas exchange characteristics did not statistically differ between the single H_2_O_2_ treatment and the control, while H_2_O_2_ positive effect was more obvious when added in combination with SDS (Fig. 3a, b, c and d).

#### Membrane damage (injury) traits and hydrogen hydroxide

**Fig. 4:**
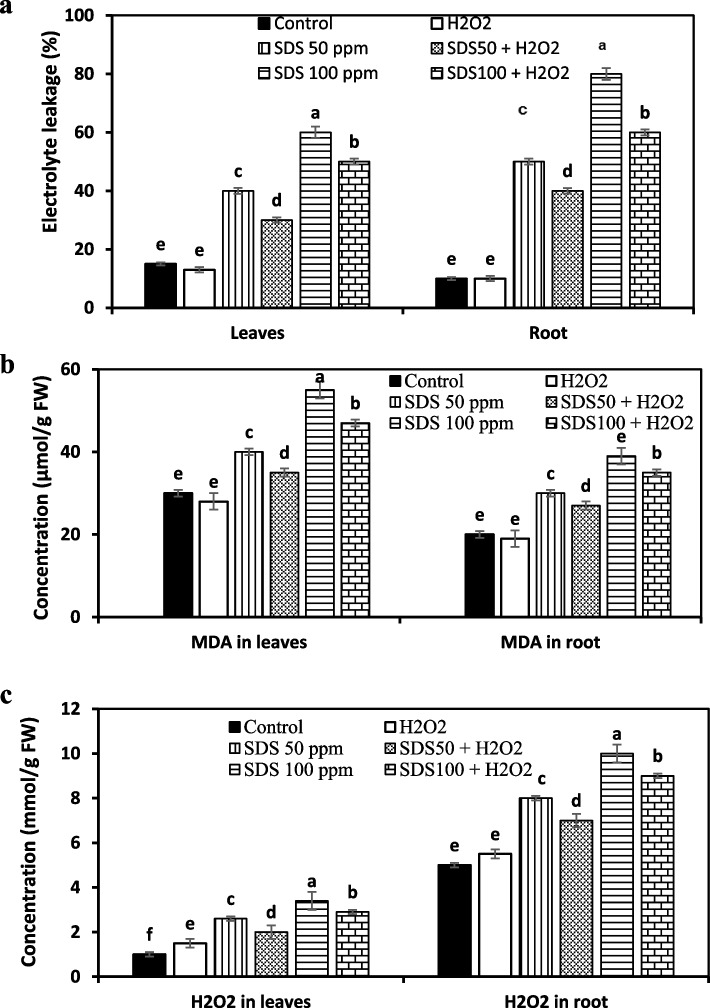
Effect of sodium dodecyl sulfate (SDS) and hydrogen peroxide (H_2_O_2_) foliar spraying on electrolyte leakege (**a**), hydrogen peroxide (H_2_O_2_) (**b**), and malondialdehyde (MDA) (**c**) concentrations of leaves and root *Juncus acutus* grown in solution culture with increasing SDS concentrations (0, 50 and 100 ppm) treated or not with 15 mM H_2_O_2_. Values are the means of four replications ± SE. Variants bearing the different letters donate statistically significant at *P* > 0.05

Electrolyte leakage (EC), malondialdehyde (MDA) and hydrogen peroxide (H_2_O_2_) contents were analysed to assess the oxidative damages in leaves and roots of *J. acutus* as affected by SDS and H_2_O_2_ treatments. Results showed sharp increase in EC values by 2.6 and 4-fold in leaves and by 5 and 8-fold in roots under 50 and 100 ppm SDS, respectively. These attributes improved more under H_2_O_2_ to lessen to 2 and 3.3-fold in leaves and by 4 and 6-fold in roots under 50 and 100 ppm SDS, respectively (Fig. 4a).

The same trend was noticed when SDS at both doses (50 and 100 ppm) caused marked rise in MDA levels in leaves and roots of plantlets, while H_2_O_2_ pretreatment considerably mediated SDS effect. In comparison to SDS treated plant, H_2_O_2_ resulted in 12.5 and 14.5% decrease in MDA level in leaves and 10 and 10.3% in roots in response to 50 and 100 ppm SDS, respectively. (Fig. 4b).

Importantly, H_2_O_2_ production drastically boosted by increasing SDS level in the culture media, but external addition of low H_2_O_2_ dose with SDS at 50 and 100 ppm positively reduced the harm effect of SDS by 23.1 and 14.7% in leaves and by 12.5 and 10% in roots, respectively, compared to SDS treated plants (Fig. 4c).

#### Metabolites and total antioxidants

**Fig. 5:**
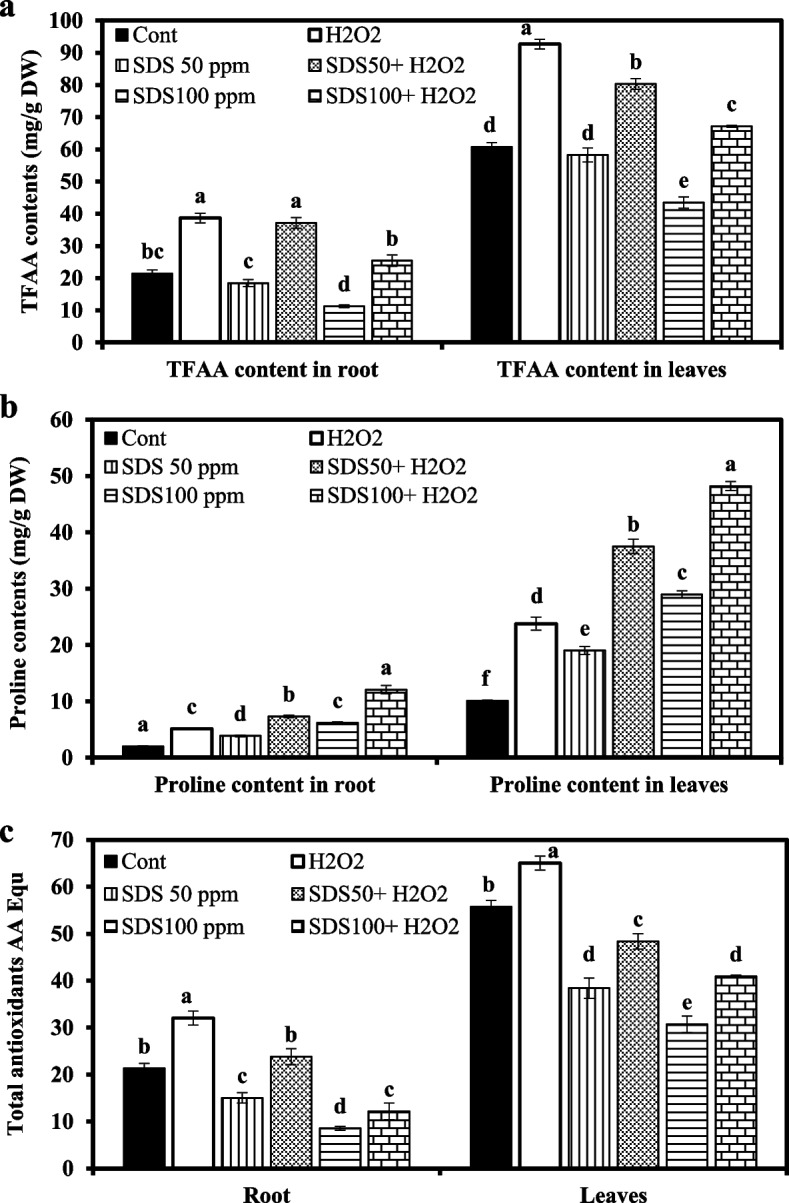
Effect of sodium dodecyl sulfate (SDS) and hydrogen peroxide (H_2_O_2_) foliar spraying on total free amino acid (TFAA), proline, and total antioxidant concent of root and leaves *Juncus acutus* grown in solution culture with increasing SDS concentrations (0, 50 and 100 ppm) treated or not with 15 mM H_2_O_2_. Values are the means of four replications ± SE. Variants bearing the different letters donate statistically significant at *P* > 0.05

Total free amino acids (TFAA) of leaves and roots were negatively influenced with increasing levels of SDS. Combined application of H_2_O_2_ and SDS (50 and 100 ppm) significantly enhanced the level of TFAA synthesis by 101.5 and 126% in roots and by 37.8 and 54.53% in leaves, respectively, over SDS contaminated plants. Moreover, sole H_2_O_2_ treatment was optimum to achieve higher generation of TFAA compared with the control (Fig. 5a).

Total antioxidant contents of *J. acutus* significantly decreased with SDS treatments as compared to control plants (Fig. 5b). Involvement of H_2_O_2_ significantly amplified total antioxidants values to rise by 58.6 and 42% in roots and by 25.9 and 33.2% in leaves under 50 and 100 ppm, respectively, as compared to respective SDS treated plants.

In contrary, proline exhibited an obvious increase due to SDS treatments as compared to control. However, H_2_O_2_ pre-supplementation (either with or without SDS) further significantly increased proline content (Fig. 5c). In roots, proline concentrations increased in the plantlets pretreated with H_2_O_2_ by 89.9 and 95.6%, while in leaves, increased by 97.2 and 66.4% at 50 and 100 ppm SDS, respectively (Fig. 5c).

### Enzymatic antioxidants

**Fig. 6:**
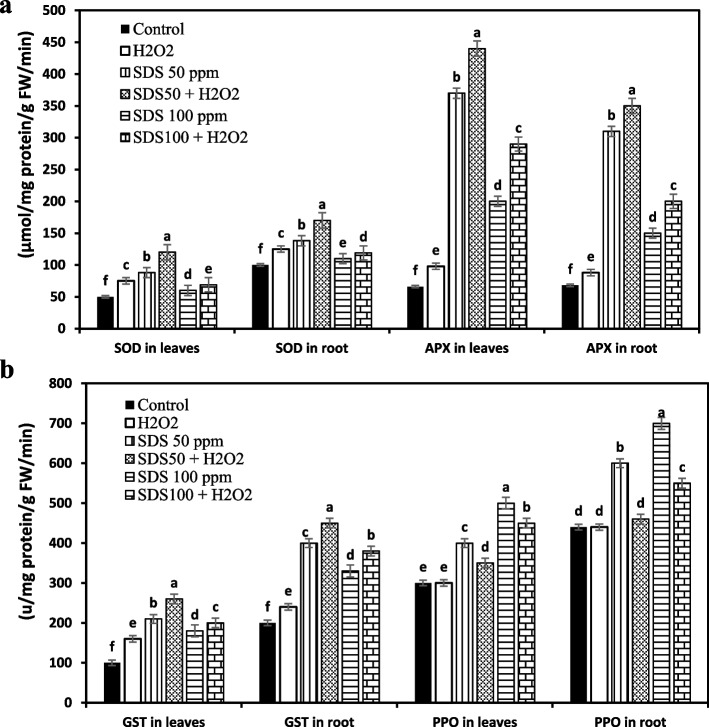
Effect of sodium dodecyl sulfate (SDS) and hydrogen peroxide (H_2_O_2_) foliar spraying on alternations in the capacities of enzymatic antioxidant: superoxide dismutase (SOD) and ascorbate peroxidase (APX) (**a**), and glutathione-s-transferase (GST) and polyphenol oxidase (PPO) (**b**) of *Juncus acutus* leaves and root grown in solution culture with increasing SDS concentrations (0, 50 and 100 ppm) treated or not with 15 mM H_2_O_2_. Values are the means of four replications ± SE. Variants bearing the different letters donate statistically significant at *P* > 0.05

As Fig. 6 shows, the activities of antioxidant enzymes; SOD, APX, GST and PPO markedly increased with increasing levels of SDS in the growth medium. Obliviously, H_2_O_2_ application was able to induce over-accumulation of antioxidant enzymes in leaves and roots of *J. acutus* in stressed and unstresses plants. Joint application of H_2_O_2_ with SDS (50 ppm) promoted SOD, APX, and GST activities by 1.4, 4.5, 1.2-fold in leaves and by 1.2, 1.1 and 1.1-fold in roots, respectively. However, H_2_O_2_ combined with SDS (100 ppm) persuaded 1.1, 1.5 and 1.1-fold augmentation in leaves, and 1.1, 1.3 and 1.2-fold increase in roots for SOD, APX, and GST, respectively. On the other hand, PPO activity in SDS treated plants was quite high and showed curtail in levels after the addition of H_2_O_2_ and SDS (50 and 100 ppm).

## Discussion

The increased surfactants consumption and discharge above threshold levels seems to be dangerous risk adversely affect the biota and all living organisms. High concentrations of SDS contaminant may cause menacing effects on plants as earlier reported [9–11]. In the current study, we noticed that *J. acutus* cleaned up SDS from the grown media and accumulated high levels of SDS in roots and leaves. The extraction efficiency could be due to the potential of *J. acutus* to tackle xenobiotic contaminants from the soil and partition them through different plant parts [26]. Similarly, SDS accumulated by numerous phytoremediators with variable mechanisms. Forni et al. [32] found that *Lemna minor* L. and *Azolla filiculoides* Lam. removed SDS from the water and accumulated it in high levels with different tolerability and accumulation patterns, as *L. minor* bioaccumulated the SDS, while *A. filiculoides* biodegraded it, particularly at lower levels. As we found here, *J. acutus* was former reported to uptake Zn from rhizosphere to roots and then translocate it into stems in mine polluted sites [29]. In this study, phytoremediation powerful seemed to be augmented when plants were pretreated with hydrogen peroxide. The application of H_2_O_2_ remarkably improved SDS removal from the culture media and assisted in increasing the bioaccumulation and translocation of the pollutant inside the plant without showing toxic symptoms. This exhibits the astonishing effects of H_2_O_2_ in remediating SDS pollutant. Plant species with BCF or TCF of value ≥1 are considered hyperaccumulators. The increase in accumulation and translocation factors reflects the advanced phytoremediation potential of a plant, which achieved in our study. The greater ability of *J. acutus* to remove SDS from the media is coupled with the better biomass production and efficient growth in the presence of H_2_O_2_. Hydrogen peroxide at certain levels has been shown to play a vital role in plant metabolism and involved in multifarious responses to environmental stresses [33].

SDS reduced plant biomass as revealed by stress tolerance index. PHSI, PDSI, and PFSI were negatively responded to SDS exposure. The plant’s biomass rate is an early indicator in stress signaling when plant exposes toxicity endpoint. However, *J. acutus* individuals survived upon exposure to SDS, they exhibited a reduced biomass compared to unstressed plants, which reveals stress disarrays entailed by SDS. These results were particularly observed in wheat and cucumber when plants were subjected to 13 different polar heads of dodecyl surfactants (10 up to 1000 mg/L) and showed significant decline in germination rate and consequence shoot and root development, especially at higher concentrations [34]. The same negative impact was recognized on several plant species [35]. This probably happens because sodium ions and/ or sulfate in SDS cause toxicity disorders in plants growth. High Na^+^ levels impede protein synthesis and intercede with enzyme regulation in plants [36], whereas sulfate in high levels negatively affects plant growth by interfering with the plant’s intercellular components [11]. In our work, H_2_O_2_ pretreatment reduced the negative impact of SDS by showing enhancement growth in stressed plants, although plants absorbed and accumulated higher concentrations of SDS. Earlier studies showed that H_2_O_2_ pre or post supply donates to the improvement of plant growth under several stresses [37, 38]. This effect may be due to the specific contribution role of H_2_O_2_ in promoting cellular proliferation and differentiation [39], up-regulating endogenous plant hormones and modulating antioxidants capacity under stress [20–40].

Our findings about decline in plant biomass and stress tolerance index directly associated with significant decrease in photosynthetic pigments and gas change attributes due to the close relation between the essential and ubiquitous photosynthetic pigments and plant growth criteria [41]. This inhibitory effect of surfactants on photosynthetic characteristics is confirmed by previous reports [42, 43]. Surfactants were proven to prompt impairment of light-harvesting pigments by inhibiting pigment biosynthesis through disturbance of protein manufacturing [42] or by weaken pigment/protein complexes, which accelerate the rate of pigments degradation [44]. Moreover, surfactants may suppress enzymatic activities which regulate stomatal closure and related gas exchange and CO_2_ fixation, this in turn restrict cell division and expansion that was evident in the current study by reducing water use efficiency, transpiration rate, and net assimilating rate. The modulation mechanism brought by H_2_O_2_ pretreatment in stressed plants which were noted in our investigation could be due to the restoration of antioxidants homeostatic balance. H_2_O_2_ is an important protective molecule can lower the adverse activity of ROS and then protect the chloroplast machinery. Nazir et al. [45] reported the ameliorative impact of H_2_O_2_ (0.1 mM) in eliciting stomata opening which were misfunctioned in copper stressed *solanum lycopersicum*. It is also stated that H_2_O_2_ may increase the optimum carboxylation rate of Rubisco and initial Rubisco activity through regulating the Rubisco enzyme [46]. These evidences match our results that H_2_O_2_ at low dose acts as a signaling molecule contributes in improving photosynthesis dynamics and stomatal opening which in result increased water use efficiency and then net assimilation rate which all this could promote sustainable biomass.

In our exploration, SDS triggered membrane injury as influenced electrolyte leakage (EC), malondialdehyde (MDA) and hydrogen peroxide parameters. SDS initiated rapid increase in EC percentage in folds over control in conjunction with high induction of oxidative damage (i.e., MDA and H_2_O_2_ concentrations) in leaves and roots. This is in agreement with numerous studies indicated the negative impact of surfactants on depolarizing the plant cell membrane [35], which probably due to the harmful impact of SDS on binding active cell wall molecules leading to a leakage of intracellular apparatuses and cell apoptosis [10]. Nevertheless, pretreatment of H_2_O_2_ was effective in protecting from the SDS stress-induced oxidative damage and membrane destruction by subduing the evoke of MDA and H_2_O_2_ at cellular and organ levels. These results are in consistent with those of [47, 48], who signified that the H_2_O_2_-inducible decline in MDA and H_2_O_2_ production seems to be one of the central features that helped to decrease cell injury and membrane integrity leading to oxidative stress downregulation.

Under harsh conditions, the accumulation of metabolites such as free amino acids and proline is well established to extend plant withstanding to stress. Amino acids are signaling molecule that act as precursors for various plant metabolites and protect cellular functions in response to multiple stresses [49]. Proline is a type of amino acids contribute to maintain plant sustainable growth and adaption under stress [22–51]. When plants face harmful stress, proline amasses in great folds to protect the cellular osmotic balance and protein stabilization, which contribute to ROS intermediation [52]. One of the adaptive mechanisms of plants to maintain their functional equilibrium is to produce significant amounts of amino acids. For that reason, the increase in metabolites production such as amino acids and proline is usually translated into increase in plant survival and adaption against severe stress [53]. At the end of the experiment, we noted that SDS at both levels induced the overaccumulation of proline in the leaves and roots of *J. acutus* and inhibited amino acids production. Loading the negative charge of surfactants to amino acids causes misfolding and denaturation of proteins, thus reducing protein production and function [54]. It was also observed that meltabilities accumulation in the H_2_O_2_ supplemented plants was sequentially increased and was positively associated with *J.acutus* tolerance upon SDS exposure in compared to SDS- stressed and unstressed plants. Besides, H_2_O_2_ also increased total antioxidant quantities in significant way in plant leaves and roots compared to SDS stressed plants without H_2_O_2_, which could be due to the improved activity of antioxidant enzymes.

Higher plants developed an antioxidant defense mechanism that mediates oxidative stress. Apart from the defense mechanism, are antioxidant enzymes that detoxify deleterious effects of ROS and strengthen the acclimization of plants under stressful conditions. The antioxidant enzymes, i.e., SOD, APX, GST, and PPO protect cellular damage and prevent the excessive formation of ROS. In the absence of H_2_O_2_, SDS at both levels increased the production of antioxidant enzymes in varying levels between leaves and roots comparing to controls. The possible elucidation might be that the plant stimulates antioxidant enzyme activities to improve the basal antioxidant energy required to endure oxidative stress. Our findings extend the same trend observed in *Azolla filiculoides* Lam., when expressed high levels of APX, CAT, PPO, and POD after exposure to sodium dodecyl benzene sulfonate (SDBS) anionic surfactant for 3 and 7 days [36]. Notably, the outcomes of our work reveal a higher level of antioxidant enzymes activities in *J. acutus* whose individuals were pretreated with H_2_O_2_ than those of SDS-treated plants only. It appears that H_2_O_2_ improved the activities of the various enzymes presented in the investigation which might further diminish oxidative damage. In specific, SOD is a key enzyme, rapidly evokes under stress conditions and has an energetic role to dismutase superoxide (O_2_^•−^) to non-toxic molecules in cells, was improved by H_2_O_2_. Boosted oxidative stress resistance due to increased SOD overproduction by H_2_O_2_ supplementation has also been clarified before in serval studies [55–57]. Likewise, extended APX activity as a result of H_2_O_2_ supply (50 μM), was shown to improve *Brassica juncea* L. stability against elevated levels of nickel stress [58]. Positive correlation was found between increased activity of APX and the ability of pistachio leaves to lower endogenous H_2_O_2_ under salt stress after 1 mM H_2_O_2_ treatment [37]. Regarding GST, its activity raised under H_2_O_2_ in the current examination, and this is in line with the results of [59], where GST is involved in downregulating ROS production under heavy metal stress. Interplay between H_2_O_2_ and PPO in the oxidative response of plants to cold stress on tomato plants was detected [40]. Interestingly, PPO displays different trend in the current examination where its activity was significantly lessened by H_2_O_2_ treatment. PPO has been proposed to play an vital role in the lignification of cell walls material that highly deposited as a mechanical barrier against external stressors, e.g. heavy metals and xenobiotics [60], preventing their entrance but in the other hand restricting normal cell elongation that witnessed in the current examination by the stunted plants, however, H_2_O_2_ treatment showed the ability to reduce PPO activity and subsequent adequate lignification regulated from high inducible rate (restricting cell growth) to relatively low or moderate level to the extent that permits cell elongation and growth [60].

Interestingly, expression levels of SOD, GST, and PPO activities in roots were greater than those in leaves; while APX activity was higher in leaves compared to roots. This effectual scavenging mechanism may be because of the defense potential of root system of *J. acutus* to phytoextract high levels of SDS as revealed by high bioaccumulation factor without showing severe damage.

Taken together, Hydrogen peroxide neutralized SDS stress-induced ROS generation by activating the enzymatic antioxidants defense mechanism, which in turn stabilized redox homeostasis and protected cellular functions. This behavior can be proved by the lower accumulation levels of H_2_O_2_ in leaves and roots of plants pretreated with H_2_O_2_, revealing improved resistance under SDS stress. Hydrogen peroxide could drive the decline in biochemical attributes in SDS-affected plants.

## Conclusion

Within the concept of phytoremediation, the results of the present study suggest that *J. acutus* could serve as a potential phytoremediator in SDS contaminated sites with variable accumulation degrees between plant organs. *J. acutus* can accumulate and tolerate considerable levels of SDS in polluted culture media, which distinguishes hyperaccumulator plants. Hence, it could be exploited as a promising biomarker for SDS pollution. However, the greatest effectiveness of SDS removal from media was obtained through H_2_O_2_ presence. Hydrogen peroxide has an enhancer effect in phytoremediation process. Pretreatment of plants with low level of H_2_O_2_ promoted SDS-stress responsive mechanisms that limited the adverse effect of SDS on *J. acutus* growth and allowed more SDS accumulation in plant tissues. We provide persuasive evidence on the enhancement role of H_2_O_2_ by the observed increase in plant biomass and, pigment contents, gas exchange attributes performance, total free amino acids accumulation, proline content, total antioxidants capacity, activities of antioxidant enzymes, low MDA and H_2_O_2_ accumulation accompanied by reduction in membrane damage. Together these effects assisted in modulating oxidative stress of SDS and improving phytoremediation potential. We suggest that integrated application of H_2_O_2_ in future phytoremediation strategies should be considered as a key factor could improve phytoremediation powerful.

## Materials and methods

### Plant growth and experimental design

The trial was conducted at Botany and Microbiology Department, Faculty of Science, Assiut University, Egypt. In the current research work, donor material of consistent grown *J. acutus* plantlets (about 35 days old, 25 g fresh weight and 15–18 cm high) were procured from the Center of Desert Agriculture in Assiut University, Egypt. *J. acutus* sample was pressed using firm cardboard sheets as an herbarium voucher specimen deposited in the botanical herbarium of Assiut University, Egypt. The species was authenticated by Prof. Momen Zareh, Professor of Plant Taxonomy and Flora at Botany and Microbiology Department, Faculty of Science, Assiut University, Egypt. For the trail, roots were finely washed under running tap water to remove the soil debris then were transplanted into clean glass containers containing gravel as a substrate and covered with aluminum foil to minimize water evaporation. Plants were initially irrigated with Hoagland nutrient solution and then irrigated once a week to provide the same quantity of water for all plants as described by Christofilopoulos et al. [28]. After 4 weeks of acclimatization in a greenhouse, plants were foliar sprayed with 30 ml of H_2_O_2_ (15 mM) three times daily for two days and left in a normal chamber for 12 h to allow the absorption of the H_2_O_2_ solution. The underground parts of plants were well covered to avoid the spray of H_2_O_2_. SDS (purity > 99%) which obtained from Sigma-Aldrich (St. Louis, MO, USA) was added to the solution culture in rate of 50 or 100 ppm. Plants were then separated into 6 treatment groups: (1) the control group, where the plants were foliar sprayed with water and grown in Hoagland solution (2) the H_2_O_2_ group, where the plants were pretreated with foliar spray of H_2_O_2_ (15 mM) (3) the SDS group, where the plants were treated with the lower concentration of SDS (50 ppm) (4) The combined H_2_O_2_ and SDS group, where the plants were foliar sprayed with H_2_O_2_ under 50 ppm SDS (5) the SDS group, where the plants were treated with the higher concentration of SDS (100 ppm), (6) the same as fourth group but under increased SDS concentration (100 ppm). The final SDS concentration of the nutritive solutions was tested as described in SDS assay section that comes below. Two foliar spray treatments (water, H_2_O_2_) were applied using hand pump trigger sprayers [60]. The containers were arranged in a completely random arrangement with four replicates and were covered by Aluminum foil keeping roots in dark conditions to prevent the algal growth. The harvest schedule of the plants was set up to be on the 15th day of SDS treatment. During harvest, the plants were gently removed from the containers and separated into leaves and roots, then carefully washed. The measurements of fresh and dry weights (g), and heights (cm) were obtained from randomly selected plants from each treatment, then collected plant parts were cut into small pieces and sampled. Fresh samples were used for chlorophyll assessment and electrolyte leakage determination, while additional samples were instantly snap-frozen in liquid nitrogen and quickly transferred to – 80 °C in the lab for detailed enzymatic and biochemical analysis. The remaining material was dried in a 70 °C oven for 48 h and weighed, then crushed to be readily for use.

### SDS assay

In reference to the methodology of Hayashi [61]**,** SDS analysis was pursued in plant samples and growth media by Methylene Blue Active Substrate (MBAS) protocol**.** SDS level was assessed by the methylene blue colorimetric assay at wavelength is 655 nm with sensitivity of 0–6 μg of SDS against pure chloroform as a blank sample. The extinction coefficient at 655 nm is of the SDS-methylene blue salt.

### Accumulation and translocation of SDS

The bioaccumulation factor (BCF), translocation factors (TCF), and % Removed SDS as described by [23–62] were applied to evaluate the phytoextraction efficiency of plants as follow:

Bioaccumulation factor: SDS concentration in the roots/SDS concentration in medium.

Translocation factor: SDS concentration in the leaves/SDS concentration in the roots.

% Removed SDS: SDS uptake by root/Added medium SDS.

### Growth stress indices parameters determination

At the end of the experiment, from the obtained data of lengths, and fresh and dry mass of plantlets, the stress tolerance index was calculated. The plant length stress tolerance index (PHSI), plant dry matter stress tolerance index (PDSI), and fresh matter stress tolerance index (PFSI) were determined according to Nawaz [63] as follow: -.

**PHSI (%)** = [The length of treated plantlets/the length of control plantlets] × 100.

**PDSI (%)** = [Dry matter of treated plantlets/dry matter of control plantlets] × 100.

**PFSI (%)** = [Fresh weight of treated plantlets/fresh weight of control plantlets] × 100.

### Determination of pigment contents

The contents of photosynthetic pigments; chlorophyll a (Chl a), chlorophyll b (Chl b), and carotenoids were executed as formerly described by Lichtenthaler [64]. Prior to determination of leaf pigments, fresh leaves were separated from the main culm and sampled. Then, immersed in test tubes containing 5 ml of 95% ethyl alcohol and heated in water bath at 60–70 °C for 30 min. The OD of samples was recorded via spectrophotometer at 663 and 644 nm for Chl a and Chl b*,* respectively. The carotenoid concentration was also determined by using the same plant extract and measuring the absorbance at 470 nm. The final calculations for chlorophyll and carotenoid content (mg/g FW) were performed using equations based on Lichtenthaler [64].

### Transpiration rate

As specified by Bozcuk [65], transpiration rate (TP) was measured. The daily transpiration rate (TP, g day^− 1^) per container was estimated via using the volumetric method. During the analysis, the transpiration rate (TP) on day i (g), the volume (Vi) of the entire container after loss compensation on day i (g), and the volume (V_i + 1_) of the entire container before loss compensation on day _i + 1_ (g) was registered. Compensation was carried out by substituting the same lost amount of water through transpiration (i.e., TP). TP was assessed using the introduced formula:

$$\textrm{TP}={\textrm{V}}_{\textrm{i}}+{\textrm{V}}_{\textrm{i}+1}$$

### Leaf stomatal conductance

Leaf stomatal conductance was estimated adopting equation recommended by Dawood and Abeed [66] in which stomatal conductance is expressed as the reverse of the stomatal resistance. The stomatal resistance measured from the following equation which displayed by Slatyer and Markus [67] and as modified by Abeed et al. [68]

$$T=\frac{Cleaf- Cair}{r\ leaf+ rair}=\frac{0.622p}{p}x\ \frac{eleaf- eair}{rleaf+ rair}$$

where rleaf + r_air_ = r. is the total (stomatal) resistance at the leaf-air interface, then

$$r.\left(\frac{\mathit{\sec}}{cm}\right)=\frac{0.622p}{p}\times \frac{eleaf- eair}{t}$$

Where: T. = transpiration rate (mg H_2_O/cm^2^/sec), r. = total stomatal resistance (sec/cm), Cleaf = the level of water vapor in leaf (absolute humidity) (mg/cm^3^), Cair = the level of water vapor in air (mg/cm^3^), eleaf = the vapor pressure inside leaf (mm Hg), eair = the vapor pressure of air (mm Hg).

Δe = eleaf - eair is the difference in vapor force between leaf and air bulk outside. The value 0.622 p/p. is a constant conversion factor to modify from Δc (cleaf- cair) to Δe. It has a value of nearly 10^6^, so 1 mm of vapor force is equivalent to about 1 mg of water vapor for each liter of air. In the case of most of stomata are on one leaf side, r. will vary markedly for the upper and lower surfaces [69].

### Water use efficiency (WUE)

For water use efficiency estimation, the containers were checked for water loss by measuring the level of the liquid medium in each container prior to every compensation time, and the differences in volumes were converted from ml to kg. The obtained measurements for each container revealed the volume of water applied to the container at that period. The water use efficiency according to Larcher [70] was determined as follows:

WUE (g/kg) = Biomass (mg DW)/ H_2_O loss.

### Net assimilation rate

Net assimilation rate was determined as applied by Dawood et al. [71] according to the following formula:

Net assimilation rate = (ln LDM_1_ − ln LDM_2_)/ [(t_1_ − t_2_) × LA_2_] g/cm^2^/d.

LDM_1_, _2_ and LA_2_ are the dry weights of leaf and the leaf area recorded before (t_2_) and after (t_1_) treatment, respectively.

### Electrolyte leakage

Electrolyte leakage (EC %) was assessed following the procedure of Abeed and Dawood [72]. For this, healthy fresh samples of leaves and roots were washed with deionized water and cut into small pieces and, then soaked in 30 ml of deionized distilled water at 10 °C. After 24 h, the elementary electrical conductivity (C_1_) of the bathing solution was noted at 25 °C. Then, leaf discs were autoclaved for 15 min and left to cool down to 25 °C and the secondary electrical conductivity (C_2_) was reported.

EC was evaluated in percentage via the following formula:

$$\textrm{EC}=\left({\textrm{C}}_1/{\textrm{C}}_2\right)\times 100$$

### Lipid peroxidation

The accumulation of malondialdehyde (MDA), a product of lipid peroxidation, was evaluated by the scheme of thiobarbituric acid (TBA) and the contents of MDA in cell membranes were determined as stated previously by [72]. First, tissue segments were accurately weighed and stabilized in 0.1% trichloroacetic acid (TCA) and then centrifuged for 10 min at 10,000 rpm. Next, 1 ml of the aliquot was mixed with TCA-TBA reagent. Finally, the mixture was heated on water bath at high temperature (95 °C) for 30 min, then cooled quickly in an ice-bath, followed by centrifuging at 10,000 rpm for 15 min and the absorbance was observed at 532 nm. Calculations were adjusted for unspecific turbidity by subtracting the absorbance at 600 nm and the results expressed as μmol/g FW [73].

### Hydrogen peroxide (H_2_O_2_)

H_2_O_2_ levels in Juncus leaves and roots was quantified as reported by Mukherjee and Choudhuri [74]. Briefly, test materials (0.5 g) were completely extracted in 4 ml cold acetone. Three ml of the acetone extract was added to 1 ml of titanium dioxide (0.1%) in 20% H_2_SO_4_ and the two mixtures were centrifuged together at 6000 rpm for 15 min. The resultant yellow color of the reaction was read spectrophotometrically at 415 nm.

### Total free amino acids

The framework of Moore and Stein [75] was used for the estimation of total free amino acids (TFAA). After accurate extraction of samples and analytical treatment with different chemicals conceded in the protocol, TFAA content was calculated from a calibration curve using glycine as blank and the wavelength was recorded at 570 nm. the data were expressed as mg/g DW.

### Proline content measurement

The extraction of proline was performed using the protocol of Bates et al. [76]. In test tubes, fine powdered dry samples were fully macerated in 3% sulfosalicylic acid and a prepared mixture solution containing proline, glacial acetic acid and acidic ninhydrin (1: 1: 1, v/v) and boiled for one hour at 100 °C. The reaction was terminated by placing the tubes in an ice bath. Then the reaction mixture was extracted with toluene (2 ml), mixed via vortex. Using toluene as blank, the optical density of the organic phase was taken at a wavelength of 520 nm.

### Total antioxidant

The method of Prieto et al. [77] was applied for the assay of the total antioxidant. Alcoholic extract with reagent mixture of 0.6 M sulfuric acid combined with 28 mM sodium phosphate and 4 mM ammonium molybdate; were well mixed and incubated at 95 °C for one hour and half, and then the mixture was allowed to cool down at room temperature. The absorbance of the mixture was observed at 695 nm and the content of total antioxidants was estimated from its standard curve.

### Enzymatic antioxidants

Twenty milligrams of frozen juncus samples were crushed to a fine powder with liquid N_2_ and then smoothed with 3 ml of 100 mM potassium phosphate buffer at pH 7.8, containing 0.1 mM ethylenediamine tetraacetic acid (EDTA) and 100 mg polyvinylpyrrolidone. The suspension was centrifuged at 18,000 rpm for 10 min at 4 °C and the supernatants collected and used for the assayed superoxide dismutase, catalase, peroxidase, and ascorbate peroxidase. All colorimetric measurements were performed at 20 °C via UV spectrophotometer [17].

Superoxide dismutase (SOD) activity was determined as documented by [17]. The activity of SOD (EC 1.15.1.1) was measured in assay mixture (2 ml), which included 100 μl enzymatic extract treated in 50 mM of sodium carbonate buffer (pH 10.2), 0.1 mM EDTA and 100 μl of 5.5 mg/ml epinephrine (liquified in 10 mM HCl, pH 2). Reads were registered by using UV spectrophotometer at 480 nm for 1 min. The SOD activity was expressed in μmol/mg protein/g FW/min. The assessment of ascorbate peroxidase (APX) activity was conducted spectrophotometrically following the steps in the protocol of Abeed et al. [17]. The activity of (APX; EC 1.11.1.11) was evaluated by the oxidation rate of hydrogen peroxide–dependent of ascorbic acid in a reaction mixture of 50 μl enzyme extract added to potassium phosphate buffer (50 mM, pH 7), Na_2_-ETDA (0.1 mM), and H_2_O_2_ (5 mM). The oxidation rate of ascorbic acid was estimated from the decrease in absorbance at 290 nm for 1 min. For measuring polyphenol oxidase (PPO) activity, mix of phosphate buffer (0.1 M at pH 6.0), catechol (0.1 M) and enzyme extract (0.5 mL) was retained in 25 °C for 5 min, then the reaction was end by adding 1 mL sulfuric acid (2.5 N). The change in absorbance was read at 495 nm and expressed per mg protein per minute [78]. For glutathione-S-transferase (GST), (GST; EC 2.5.1.18, u/mg protein/g FW/min) was quantified by following the methods adopted by AbdElgawad et al. [79].

### Statistics

All values given in this trial are average of four samples, presented with standard deviation. The descriptive statistics to determine the significant differences between treatments were investigated by analysis of variance (ANOVA) by SPSS 21.0 software at 5% level of probability. Mean values for the treatments were compared using Duncan’s multiple range test.

## Data Availability

All data generated or analysed during this study are included in this published article. The data will be shared on reasonable request of the corresponding author.

## Abbreviations

SDS: Sodium Dodecyl Sulfate
H_2_O_2_: Hydrogen peroxide
FW: Fresh weight
DW: Dry weight
TFAA: Total free amino acid
MDA: Malondialdehyde
ROS: Reactive oxygen species
SOD: Superoxide dismutase
APX: Ascorbate peroxidase
GST: Glutathione-s-transferase
PPO: Polyphenol oxidase
